# The Relationship Between Rape Myths, Revictimization by Law Enforcement, and Well-Being for Victims of Sexual Assault

**DOI:** 10.1177/10778012231196056

**Published:** 2023-08-21

**Authors:** Nicole Maiorano, Áine Travers, Frédérique Vallières

**Affiliations:** Centre for Global Health, 8809 Trinity College Dublin, Dublin, Ireland

**Keywords:** revictimization, rape myths, police, sexual assault response

## Abstract

Relationships between rape myths, revictimization, and postassault well-being were examined in a sample of adult victims of sexual assault (*n* = 88). Correlation, multiple regression, and path analyses investigated whether conformity to stereotypes of “real rape” or “real victim” was associated with revictimization and well-being. A possible mediating effect of revictimization on the relationship between rape myth conformity and well-being was assessed. The relationship between specific revictimization behaviors and emotions was also analyzed. Questioning victims’ resistance to the assault was correlated with revictimization emotions. “Real victim” characteristics were associated with well-being, but no mediating effect of revictimization was observed.

Rape myths are attitudes and beliefs about rape that are widely and persistently held by individuals within a given cultural context. Early definitions described rape myths as “prejudicial, stereotyped, or false beliefs about rape, rape victims, and rapists” that may “deny or reduce perceived injury or to blame the victims for their own victimization” (Burt, 1980, p. 217). More recent definitions conceptualize rape myths as “a general cognitive schema that enables negative attributions to be made about the crime of rape and those involved” (Hine & Murphy, 2017, p. 1). Specifically, these schemas include characteristics of the victims and perpetrators of sexual assault along with elements of the act itself (Hine & Murphy, 2017).

A common rape myth is that certain sexual violence constitutes “real rape.” The notion of “real rape” is comprised of a set of commonly held stereotypes regarding the circumstances and environment in which the rape act occurs, including characteristics of the perpetrator (DuMont et al., 2003). While there is no complete list of what characteristics constitute a “real rape,” in many Western contexts, the idea of “real rape” is related to the belief that rapes occur in an outdoor setting and at night, whereby the perpetrator is unknown to the victim and uses a weapon and whereby the victim resists and is thus left with physical and emotional indicators of the struggle that ensued (Horvath & Brown, 2009; Weis & Borges, 1973; Williams, 1984). Table 1 summarizes the common beliefs associated with real rape, as identified from the extant literature.

**Table 1.** Circumstances Associated with “Real Rape.”

Circumstance	Source
Physical force used by perpetrator during assault	DuMont et al., (2003); Hockett et al., (2016)
Victims were physically harmed during assault	DuMont et al., (2003); Hockett et al., (2016); Weis & Borges (1973); Williams (1984)
Indicators of victim's physical harm (e.g., bruises, and fracture) were documented after the assault	DuMont et al., (2003); Hine & Murphy (2017); Hockett et al., (2016)
Victims did not know the perpetrator (i.e., the perpetrator was a stranger)	DuMont et al., (2003); Hine & Murphy (2017); Hockett et al., (2016); Spohn & Horney (1996); Weis & Borges (1973); Williams (1984)
Penetration occurred	DuMont et al., (2003)
A weapon was present or threatened	Hine & Murphy (2017)
The assault occurred in a public setting	Hockett et al., (2016); Williams (1984)
Victim physically resisted assault	Williams (1984); Spohn & Horney (1996)
Victim verbally resisted assault	Williams (1984)

Related to the notion of “real rape” is the idea of “real victims.” Similar to real rape, a real victim is comprised of a set of commonly held stereotypes regarding the characteristics and behavior of the rape victim (DuMont et al., 2003). Like real rape, there is no complete list of what characteristics constitute a “real victim” and specifics of these myths may vary to some degree across cultures and contexts; however, the current literature identifies various factors which may be associated with ideas of “real victims.” Evidence suggests “real victims” are younger individuals, of an ethnic majority, who are single, with no documented mental health difficulties, no past reports of having been a victim of sexual violence, no drug or alcohol usage prior to the assault, and who appear emotional when reporting the incident (DuMont et al., 2003). Real victims are also conceptualized as female (Parratt & Pina, 2017; Sleath & Bull, 2012; van der Bruggen & Grubb, 2014) and heterosexual (van der Bruggen & Grubb, 2014). In contrast, a history of work in the sex industry (Frohmann, 1991) and delays in reporting the incident (Jordan, 2004; Spohn & Horney, 1996) have been suggested to be irreconcilable with the idea of a “real victim.” Table 2 summarizes common characteristics of real victims, as identified from the literature.

**Table 2.** Common Characteristics of a “Real Victim.”

Circumstance	Source
Young in age	DuMont et al., (2003)
Majority race	DuMont et al., (2003)
Heterosexual	Van der Bruggen & Grubb (2014)
Female	Van der Bruggen & Grubb (2014); Spohn & Horney (1996)
Not in a relationship	DuMont et al., (2003)
Professional employment	Page (2010)
No history of mental health difficulties	DuMont et al., (2003); Jordan (2004)
No drug or alcohol use prior to the assault	DuMont et al., (2003); Jordan (2004); Hockett et al., (2016); Spohn & Horney (1996)
No previously reported abuse/sexual assaults	DuMont et al., (2003)
No previous reports that did not lead to a conviction	Jordan (2004)
Assault reported immediately (within 24 h of occurring)	Jordan (2004); Hockett et al., (2016); Spohn & Horney (1996)
No previous employment as a sex worker	Frohmann (1991); Page (2010)

Together, the stereotypes of real victims and real rape have important implications for the way victims of sexual assault view themselves (Patterson et al., 2009) and how others view victims (Frese et al., 2004). This can ultimately impact on the treatment and recourse options available to victims in the aftermath of a sexual assault.

It is widely documented that rape negatively and extensively impacts both the psychological and physical wellbeing of victims (Campbell & Wasco, 2005). In addition to the trauma of the rape act, the reactions a victim receives to the disclosure of their assault may also influence their emotional wellbeing. Specifically, negative reactions, including placing blame on the victim, have been shown to increase the development of posttraumatic stress disorder (PTSD) symptoms (Jacques-Tiura et al., 2010) and decrease in the use of positive coping strategies (Orchowski et al., 2013). The influence of rape myths on the relationship between reactions to disclosures of assaults and PTSD has also been considered. For example, Ullman and Filipas (2001) found that being of a racial minority was associated with experiencing more severe PTSD symptoms and that this relationship was mediated by the occurrence of negative social reactions to the disclosure of sexual assault. This finding indicates that the psychological effect of rape myths on victims may be mediated by the reactions of others.

The influence of rape myths on responses to disclosure of sexual violence may particularly be felt by victims when trying to report or seek justice for the crime(s) committed against them. Specifically, research has shown that stereotypes influence how medical personnel (Campbell, 1998), investigative officers (Hine & Murphy, 2017), and jurors (Dinos et al., 2015) view victims of sexual assault. Of these, research suggests that the influence of rape myths among investigative officers may be most harmful to victims, given that the police have the initial responsibility to investigate cases and that their investigation has important implications for how the case is processed (Alderden & Ullman, 2012). Specifically, investigative officers make judgments of a victim's credibility and evidence suggests that this is influenced by victim characteristics such as profession, age, sexual history, ethnicity, as well as victims’ behaviors (Campbell et al., 2015; Patterson, 2011; Page, 2010). A 2017 systematic review of 24 articles by Sleath and Bull (2017), for example, found that elements of rape myths pertaining to victim characteristics affected how investigative officers viewed the credibility of the victim and the attribution of guilt toward the victim. This study found police officers placed more blame on victims who knew the perpetrator, were male, and were judged by the officer as provocative based on variations in the attire of the victim; mixed results were found for the influence of alcohol consumption on blame attribution. Certain characteristics, including detail and consistency in victims’ statements and the presence of physical evidence and physical injury, were also shown to increase the credibility of victims among officers. While no relationship was found between levels of blame placed on a victim by officers and how the case progressed, cases wherein the victim was judged as more credible and less responsible for the sexual assault were more likely to result in action from investigative officers. Sleath and Bull therefore conclude that elements of rape myths present in officer's judgments of victims have implications for how cases progress.

Hockett et al. (2016) propose that rape myths can result in blame being placed on victims who do not fulfill the stereotypes of a real victim having experienced a real rape. Hockett refers to the impact of this skepticism and blame as “secondary victimization,” also referred to as revictimization. Defined as a “negative social or societal reaction in consequence of the primary victimization […] experienced as further violation of legitimate rights or entitlements by the victim” (Orth, 2002, p. 314), revictimization can cause additional psychological harm to victims of sexual violence. A study of revictimization conducted by Campbell and Raja (1999) with service providers who work with sexual assault victims found that the majority of the providers felt that contact with the legal system could be psychologically harmful to victims. Additionally, Patterson et al. (2009) found that the fear of psychological harm from reporting the crime contributed to victims’ decisions to not report their sexual assault. Similarly, a United States study of 3,001 victims of rape found that of the 84.2% of those who did not report their rape(s) to the police, 42.6% stated that their decision was due to fear of the justice system (Wolitzky-Taylor et al., 2011). Thus, it appears that the fear of revictimization acts as a key barrier preventing victims from seeking justice for the crimes perpetrated against them.

The extant literature proposes an influence of rape myths on the revictimization experienced by victims (Hockett et al., 2016) and suggests that revictimization, in turn, may have compounding effects on victims’ wellbeing. While past studies of rape myths have considered elements of various myths (Hine & Murphy, 2017), to our knowledge, no studies have utilized comprehensive “real victim” and “real rape” variables to analyze the relationships between rape myths, revictimization, and wellbeing. The current study therefore aims to address this gap through three interdependent research objectives. First, we sought to identify the relationships between real rape and real victim characteristics and revictimization among survivors of sexual assault who reported their experience(s) to law enforcement. Second, we investigated which revictimization behavior(s) by law enforcement officers were most strongly associated with revictimization emotional distress. Third, we investigated whether the relationship between real victim and real rape variables and later in life subjective wellbeing (SWB) was mediated through revictimization.

Our hypotheses pertaining to the aforementioned objectives were as follows:

1. Victim and rape characteristics that more strongly align to “real rape” and “real victim” stereotypes would be negatively correlated with revictimization scores.
2. Specific revictimization behaviors by law enforcement officers would be more strongly associated with revictimization emotional distress
3. Lower revictimization experiences would attenuate the relationship between rape characteristics and long-term wellbeing.

## Method

### Participants and Procedures

Participants (*n* = 88) were between the ages of 18 and 73 years (*M*_Age_*_ _* = 34.76, *SD *= 11.64) and all identified as female. To be included in the study, the incident(s) of sexual assault had to have occurred in adulthood (i.e., ≥ 18 years old) and have been reported to the police. The participants were residents of Ireland, Scotland, Canada, England, Northern Ireland, the United Kingdom (otherwise not specified), the United States, and Australia. All participants made reports of the sexual violence in the aforementioned countries, with the addition of South Africa.

Participants were recruited through organizations that provide services to victims of crime, sexual assault, and rape, who recommended the study directly to clients, posted links to the study on their social media accounts, and/or placed an advertisement poster in their facilities. One rape crisis center in Ireland requested hardcopies of the survey, which their counselors distributed to clients who expressed an interest in participating in the study. The hardcopies were then scanned by staff at the center and electronically returned. Five participants completed the survey using this format, while the remaining participants accessed an anonymous online survey.

Upon accessing the survey, participants were presented with an information form describing the research purpose, procedures, and confidentiality. Following that, participants were presented with a consent form which required active acknowledgment (i.e., ticking a box) of electronic consent. Participants were informed that all questions and sections of the survey were optional.

The survey was not timed and allowed participants to complete it at their own pace. Each measurement tool was presented as one page of the survey for which participants could answer as many questions as they chose before continuing on to the next section. Participants also had the option to review previous sections throughout the study. After completing the survey, participants were presented with a debriefing form that briefly explained the purpose of the research and provided information for relevant support services.

### Measurement Tools

All questionnaires were presented in the order listed below. At the beginning of each section was a short description of the types of questions that would be asked and a reminder that all responses were optional.

#### Demographic questionnaire

Participants were first asked the following demographic information: current gender, age, and country of residence. Participants were also asked to report information regarding their sexual assault and/or rape including the country it was reported in, their age at the time of the assault, and the gender of the investigating officer.

#### Revictimization behaviors and emotions

Participants were next presented with a series of questions to describe their interactions with investigative officers. These questions consisted of the Revictimization Behavior and Revictimization Emotions subscales, as devised by Campbell (2006) based on her unpublished interviews conducted in 1996 with 20 rape survivors and in 1998 with 30 sexual assault community service providers on the topic of revictimization for sexual assault victims during interactions with police officers. Cronbach's alpha for the Revictimization Behavior and Revictimization Emotions subscales in the current sample were, respectively, good (α = .72) and excellent (α = .92).

The Revictimization Behavior Scale consists of 16 behaviors that may occur during interactions with investigative officers. Participants are asked to indicate whether they experienced any of these behaviors (0 = no; 1 = yes). In addition, participants indicate if their interactions with investigative officers caused or increased the eight emotions of the Revictimization Emotions Scale (0 = no; 1 = yes). The averages were calculated for both of these scales whereby a higher value indicates the experience of more revictimization behaviors or emotions.

#### Real victim

Participants were instructed that they would next be asked self-characteristics at the time of their sexual assault and/or rape. Questions were based on the work of DuMont et al. (2003), with additional questions included based on the review of literature (see Table 2). Participants were asked a single question for each of the common characteristics of a “real victim,” across a total of 12 items.

To create the “real victim” count variable, responses that aligned with the “real victim” characteristic were coded as 1, and each response that did not align with the characteristic was coded as 0. The one exception was the item age of the assault which was coded with the formula 1−(age/100) to align with the literature which purports that “real victims” are younger in age. For participants who listed multiple ages (i.e., multiple incidences), the youngest age listed was utilized in the analysis. Consistent with Table 2, participants were assigned a score of 1 if they endorsed the following rape myths: Caucasian, heterosexual, female, not in a relationship (i.e., divorced, widowed, or single), employed, did not have a history of mental health difficulties, did not use drugs or alcohol immediately prior to the assault, did not report previous sex crimes, did not make a report that did not lead to a conviction, reported the assault within 24 h, and did not previously work as a sex worker.

#### Real rape

Participants were instructed that they would next be asked characteristics of their sexual assault and/or rape. Similarly to the “real victim” scale, this scale was created based on the work of DuMont et al. (2003), with additional items identified based on the review of literature (see Table 1). Participants were asked a single question for each of the common characteristics of a “real rape.”

To create the “real rape” count variable, each response that aligned with the “real rape” characteristic was coded as a 1 and each response that did not align with the characteristic was coded as 0. The one exception was the item of relationship to the assailant. This item had four possible responses: *stranger*, *known less than 24 h*, *known more than 24 h*, and *partner or ex-partner*. The response of *stranger* was coded as 1 and the coding decreased by an increment of .25 to a score of 0 for *partner or ex-partner*. For the other variables, participants were assigned a score of 1 if the following myths were endorsed: use of physical force, physical harm to victim, documentation of physical harm, occurrence of penetration, use of physical resistance by the victim, use of verbal resistance by the victim, the use or threat of a weapon during the assault, and the assault occurring in a public setting.

#### Subjective well-being

The World Health Organization Five Well-Being Index (WHO-5) has been found to assess emotional well-being with high predictive and construct validity (Birket-Smith et al., 2009; Blom et al., 2012; Lucas-Carrasco et al., 2012). Additionally, it is recognized by clinical experts as a strong measure of well-being among the general population as well as specific groups, indicating relevance of the scale across diverse fields of study (Topp et al., 2015). This scale also demonstrated strong internal reliability among the current sample (α = .93).

The WHO-5 scale consists of five statements describing the experience of a specific positive effect (e.g., *I have felt cheerful and in good spirits*). Participants were asked to indicate the frequency of their experience of each effect over the previous 2 weeks. After each statement, participants were presented with six options (*all of the time*, *most of the time*, *more than half of the time*, *less than half of the time*, *some of the time*, and *at no time*). The responses were then coded on a scale of 0 to 5 wherein *all of the time* was coded as 5 and *at no time* was coded as 0. The five items are then used to calculate a total score, where higher scores indicate greater SWB.

### Ethics

Ethical approval for this study was granted by the University of Dublin, Trinity College.

### Data Analysis

Time since the assault occurred was calculated for each participant in years by subtracting their current age with their age at the time of the assault, as reported by participants. Mean real victim, real rape, revictimization behavior, and revictimization emotion scores were calculated for each participant by averaging the items contained within each scale. Possible scores therefore ranged from 0 to 1, wherein a higher score indicates closer alignment to rape stereotypes and greater experience of revictimization behaviors or emotions. The means were then used to test the previously outlined hypotheses.

**Figure 1.**
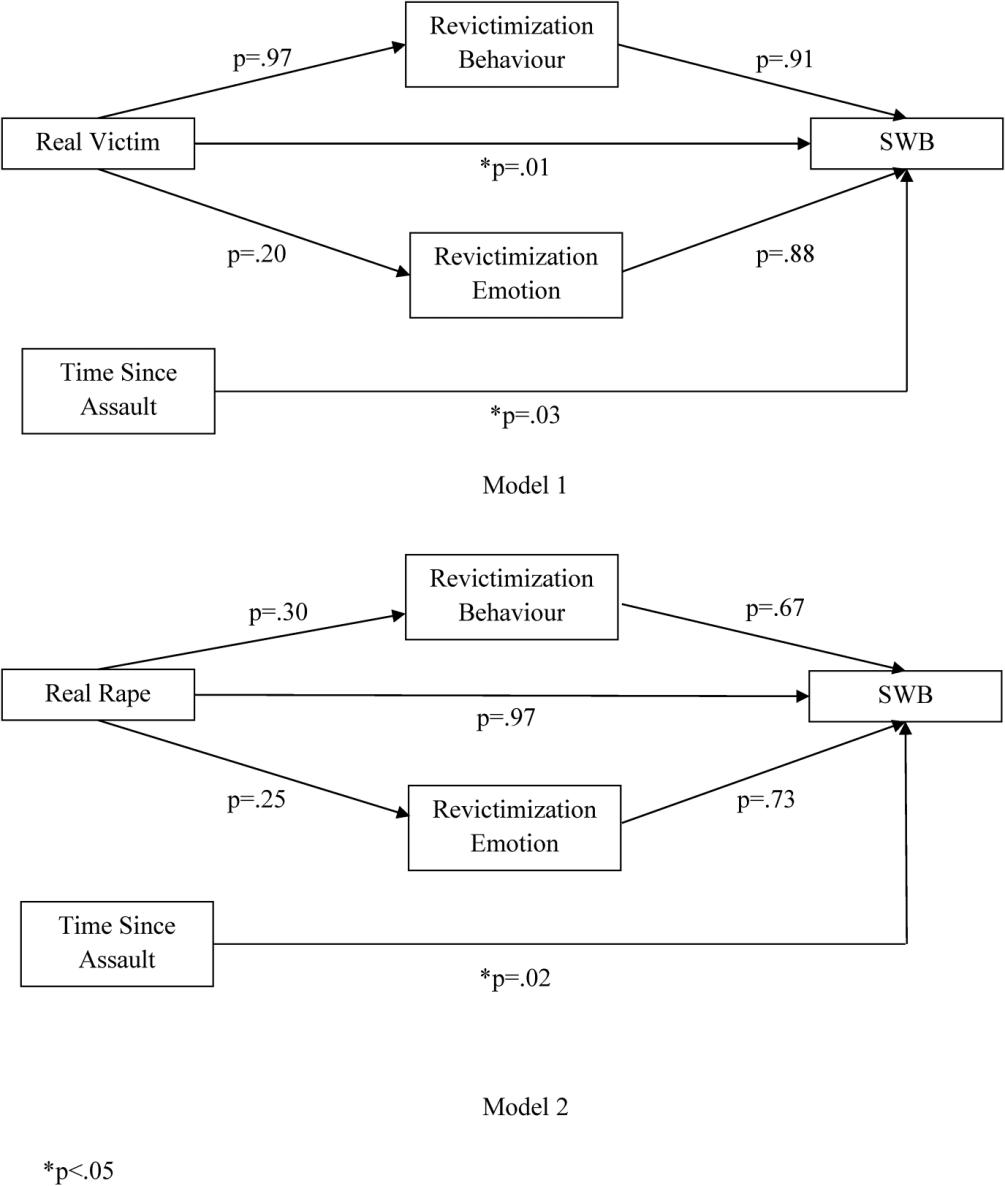
Diagrams of the Estimated Path Models.

To address the first hypothesis, Pearson's bivariate correlations were conducted to test the relationships between real victim, real rape, revictimization behaviors, and revictimization emotions. For the second hypothesis, a multiple linear regression analysis was applied to test the relationships between the individual items of the revictimization behavior scale and the mean scores on the revictimization emotion scale to determine whether, and if so which, specific behaviors were significantly associated with increased revictimization emotion scores. Finally, for the third hypothesis, two independent path analyses were conducted to test whether there was an association between the real victim and real rape variables and SWB and whether these relationships are mediated by revictimization behavior and emotion while controlling for the amount of time that has passed since the assault (see Figure 1). Analyses were carried out using SPSS Version 25, and the path analyses were carried out using the PROCESS macro (Hayes, 2017).

## Results

Participants completed the study regarding assaults that happened within the past year ranging to reports that happened 43 years ago (*M = *8.77, *SD = *9.39). Participants ranged from experiencing none of the items to all of the items of the revictimization behavior and emotion scales with a mean score of .36 (*SD = *0.20) on the behavior scale and of .68 *(SD = *0.37) on the emotion scale*.* The scores of the “real victim” scale ranged from .34 to .98 (*M = *0.78, *SD = *0.12) and the “real rape” scale ranged from .15 to 1 (*M = *0.56, *SD = *0.23). Finally, participants reported totals on the WHO-5 that ranged from 0 to 21 (*M = *10.61, *SD = *6.19).

In addressing the first hypothesis, results of the bivariate correlations suggest a significant correlation between the revictimization behavior and revictimization emotion subscales only (*r*(88) = .63, *P* < .001) (Table 3).

**Table 3.** Pearson's Correlation Between Real Rape, Real Victim, Victimization Behavior, and Victimization Emotion.

	Real rape	Real victim	Revictimization behavior	Revictimization emotion
Real rape	1			
Real victim	−.01	1		
Revictimization behavior	.04	.04	1	
Revictimization emotion	.12	−.12	.63*	1

*P < .001.

For the second hypothesis, a multiple linear regression was calculated to predict the impact of each revictimization behavior on revictimization emotion (*F*(16, 67)* *=* *3.70, *P *< .001, *R*^2^* *=* *.47). Of the 16 revictimization behavior items, the only item with a unique significant association with revictimization emotion was the item regarding the questioning of the participant's resistance to the assault (β* *=* *.21, *P *= .04). Other items such as officers refusing to take report (β* *=* *.32, *P *= .17), questioning of the participant's sexual history (β* *=* *.17, *P *= .07), and questioning of the participant's memory (β* *=* *.15, *P *= .08) did not reach statistical significance (Table 4).

**Table 4.** Multiple Linear Regression of Revictimization Behavior Items on Revictimization Emotion.

Variables	β	*SE*	*t*	*P*	95% CI
Police report created	.18	.16	1.13	.26	(−.15, .50)
Investigation carried out	.02	.12	.15	.88	(−.22, .254)
Provided referrals	.04	.08	.54	.59	(−.11, .19)
Discouraged from filing report	.01	.11	.06	.95	(−.21, .22)
Officers reluctant to take report	.05	.10	.55	.59	(−.14, .25)
Officers refused to take report	.32	.23	1.38	.17	(−.14, .79)
Said case not serious enough	.11	.09	1.21	.23	(−.07, .29)
Explain steps in reporting	.10	.08	1.24	.22	(−.06, .27)
Asked about prior relationship with perpetrator	.02	.09	.24	.81	(−.15, .19)
Manner of dress questioned	.05	.08	.63	.53	(−.11, .22)
Behaviors during incident questioned	.01	.10	.06	.95	(−.19, .20)
Sexual history questioned	.17	.09	1.84	.07	(−.02, .36)
Memory questioned	.15	.09	1.79	.08	(−.02, .32)
Resistance questioned	.21	.10	2.11	.04*	(.01, .42)
Sexual reaction questioned	−.01	.09	−.08	.94	(−.18, .17)
Asked to take a lie detector test	−.15	3.44	−.45	.66	(−.84, .53)

*P < .05

Finally, in relation to the third hypothesis, results of the mediation analysis yielded a significant effect for the direct effect of the real victim variable on SWB only, as shown in model 1 (β* *=* *15.07, s.e. = 5.86, *t*(76) = 2.57, 95% CI (3.40, 26.75), *P *= .01). In both model 1 and model 2, time since the assault also yielded a significant effect on SWB (model 1: β* *=* *.17, s.e. = .08, *t*(76)* *= 2.15, 95% CI (.01, .32), *P *= .03; model 2: β* *=* *.20, s.e. = .08, *t*(76)* *= 2.47, 95% CI (.04, .37), *P *= .02).

## Discussion

Three hypotheses were tested to understand the relationships between rape myths, revictimization, and SWB. The analyses did not support the first hypothesis that “real victim” and “real rape” characteristics, as defined in the present article, are negatively associated with revictimization scores. The only significant correlation was a positive association found between revictimization behaviors and revictimization emotions. In addressing the second hypothesis, questioning of the participant's resistance to the assault was uniquely and positively found to be associated with revictimization emotions. Finally, although a significant direct effect of real victim characteristics and of time since the assault on SWB was identified, no mediation effects as predicted by the third hypothesis were found.

Since early conceptualizations of rape myths such as Burt (1980), the theory of rape myths has grown to include multiple characteristics of acts and victims of sexual assault. Similarly, research has attempted to develop an understanding of the revictimizing nature of responses to sexual assault disclosures as illustrated by the revictimization scales used in this study. The findings indicate a direct correlation between the subscales of revictimization, providing further evidence of a connection between actions of investigators and the emotional experience of victims. The regression analysis showed only one revictimization behavior, the questioning of the participant's resistance, to be uniquely correlated with the experience of revictimization emotion. More research is needed to understand the impact of this item on the emotional experience of victims. This research can inform best practices of investigators as it may provide insight as to ways to mitigate the harm caused by this line of questioning.

While previous research (such as Campbell et al., 2015; Hine & Murphy, 2017; Patterson, 2011) has shown effects of elements of rape myths on the treatments victims receive, the current study did not find a relationship between a greater totality of these myths and revictimization. Campbell et al. (2015) concluded that investigative officers were influenced by characteristics of a victim, particularly when other forms of evidence were lacking. In cases without other forms of evidence, officers’ judgments of the victim influenced the progression of the case. This finding demonstrates the contextual nuance of these effects and the multitude of elements that affect the real experiences of victims when working with law enforcement. Accordingly, the design of the current study may have excluded mitigating contextual case factors, such as the presence or absence of corroborating evidence, that may impact the experience of rape victims.

While the path models were not supported, a greater amount of time since the assault and greater conformity to the real victim variable were found to significantly correlate with greater SWB. Multiple studies have documented high rates of PTSD for rape victims and the long-term impact of rape on physical health. (Campbell & Wasco, 2005). The relationship between time and well-being in the current data set could indicate a resilience factor of rape victims that is not fully captured by research that focuses on psychological symptomology. Previous research has conceptualized well-being as more than just the absence of psychological symptoms and demonstrated an independence of well-being as captured by the WHO-5 from symptoms of psychological disorders (e.g., depressive symptoms; Bech et al., 2006). Similarly, the current findings may support well-being as an independent construct from PTSD symptomology for rape victims. While beyond the scope of the current article, future research with rape victims should be conducted to better understand this possible distinction, recovery from symptoms, and changes in well-being over time.

The significant relationship between real victim conformity and SWB tentatively suggests a protective effect of real victim characteristics on wellbeing for rape victims. This could be a result of elements of the real victim variable being associated with well-being outside of the experience of sexual violence. For example, the real victim variable includes not having a history of mental health difficulties and not having previously experienced sexual abuse or assaults. Both of these elements may impact SWB independently of a sexual assault.

Alternatively, there may be other factors not included in this study that mediate the relationship between the real victim variable and SWB. Ullman and Filipas’ (2001) study found that being of a racial minority was associated with greater PTSD as mediated by the reactions of others to the disclosure of a sexual assault. In contrast to the current paper, Ullman and Filipas’ (2001) study included reactions from multiple sources and not specifically investigative officers. This may indicate that other factors, such as the reactions of informal support systems, may mediate the relationship between the real victim variable and SWB. Further research should aim to clarify the relationship between real victim and SWB as these findings could inform targeted interventions to improve well-being of victims.

One difficulty with understanding the current findings in relation to previous studies is the lack of a unified definition or conceptualization of rape myths and their accompanying standardized measures. As previously described, unlike the revictimization scales, the measures of “real victim” and “real rape” characteristics were created specifically for the purposes of this study. DuMont et al.’ (2003) study, which analyzed the relationship between victims’ alignment to rape myths and likelihood to report an act of sexual assault to investigative officers, provided a basis for deciding which factors to include in this measure. A further review of the literature added to their scales to create the scales used in this study. However, not all of the elements in DuMont's study were shown to have a statistically significant relationship with reporting. Specifically, the variables of age and relationship status were not significantly related to likelihood to report. DuMont and colleagues also noted that previous studies regarding the role of age in rape myths show mixed results. Therefore, while some of the factors chosen by both DuMont and the present study have been theoretically proposed to be a part of “real victim” and “real rape,” there are mixed findings in empirical studies of the relationship between rape myths and outcomes for victims. Both DuMont's study and the current study indicate a need for the development of “real victim” and “real rape” scales that demonstrate adequate convergent and discriminant validity.

Therefore, and in working toward empirical studies of rape myths and revictimization, the current study adds to the existing literature by proposing “real victim” and “real rape” variables based on DuMont et al.’ (2003) study, in addition to other theoretical and empirical works in this field. While the term “rape myths” was defined by Burt in 1980 and the terms “real rape” and “real victim” have been used throughout “rape myth” literature, to the authors’ knowledge, empirical scales for these concepts have not been created nor tested. While DuMont et al. (2003) provide a basis for this study, the review conducted in this paper expands on their definitions and provides an empirical foundation for these concepts for future research. However, a full systematic review of the literature was beyond the scope of this paper and further studies are required to validate these measures as constructs of considerable interest.

Additionally, differences in the ways data are collected and analyzed present problems for interpretation of the evidence base. For example, in a review of literature conducted by Sleath and Bull (2017), contrasting results were found in studies that analyzed the relationship between intoxication and blame placed on rape victims. DuMont et al. (2003) proposed that these contradictory results could be a result of cultural differences, shifts in views over time, or differences in the way the data were collected. Specifically, one study included questions of the perpetrator's intoxication in addition to the victim's, whereas the other focused solely on the victim's intoxication. DuMont et al. (2003) also point out that the element of age, as a component of rape myths, has been analyzed as a continuous variable and as a categorical value in different analyses. As rape myths are a reflection of societal beliefs of acts and victims of rape, it can be hypothesized that their elements and influence may change over time. However, a lack of uniformity on the study of these topics may create barriers to observing possible societal shifts, including shifts within investigative forces.

### Limitations

The current study is not without limitations. An error in the administration of Campbell's revictimization behavior scale resulted in one item (i.e., questioning by police of why the victim was with the perpetrator) mistakenly excluded from the study. Additionally, contextual limitations of the scales should be noted. The scales used were created within an urban, US context over 15 years ago, based on data collected about 10 years prior. In order to continue testing the theory of revictimization of sexual assault victims, further effort to validate these scales across contexts is necessary. This validation should consider what law enforcement behaviors may be experienced as revictimizing for victims within specific cultural, geographical, and chronometric contexts and the connection of these behaviors to revictimization emotions. However, the correlation between the revictimization behaviors and revictimization emotions subscales supports the proposal that the behaviors and emotions in the scale are positively associated within this sample of mixed nationalities.

Finally, the current sample presents possible limitations. Given the nature of the sample and the low incidence of sexual assault reporting (Morgan & Oudekerk, 2019), more generally, the current study may be underpowered. Additionally, the current sample solely consists of individuals who identify as female, and so, it was not possible to include any assessment of gender differences. Further research with larger sample sizes that include diverse gender identities is warranted to understand how rape myths and revictimization experiences may differ by gender.

### Directions for Future Research

Further research into the relationship between “real victim,” “real rape,” revictimization, and wellbeing should be conducted to better understand the translation of these theories to the experiences of victims of sexual assault. Certain design choices may be particularly important in these studies.

Specifically, the design of this study differed from previous research by directly involving the victims of sexual violence. Unlike previous studies (Campbell & Raja, 1999; Kelleher & McGilloway, 2009), the degree of revictimization was reported directly from the victims as opposed to from service providers, as recommended by Kelleher and McGilloway (2009). Additionally, previous studies that measured law enforcement's attitudes toward rape victims often used hypothetical vignettes (e.g., Frese et al., 2004; Hine & Murphy, 2017). Researchers have questioned the validity of vignettes as they may not accurately represent officers’ views (Grubb & Turner, 2012; Sleath & Bull, 2017) and may be affected by officers’ social desirability bias (Parratt & Pina, 2017). Collecting data directly from the victims avoids biases of both providers and law enforcement. Future research should build on the model of directly involving victims utilized here while increasing sample sizes to better discern the relationships between the constructs analyzed.

Additionally, the finding that the amount of time since the assault significantly correlated with SWB in both models provides support for its use in future research. Past research analyzes data from victims at varying points of time since their assault. For example, the data in Campbell's (2006) study was collected from assault victims in the hospital immediately before discharge, whereas the average amount of time since the assault of participants in the current study was ∼9 years. Designs of future research should consider the possible effect of the amount of time since the assault occurred and control for time since assault to better support intrastudy comparison. Moreover, while this finding may indicate that the negative effects of rape (Campbell & Wasco, 2005) decrease over time, additional research can explore this relationship further.

Finally, future research may benefit from the use of qualitative or mixed-methods designs. Campbell and Wasco's (2005) review of sexual assault studies concluded that qualitative research may “shed a new light on old problems.” While theories of rape myths and revictimization have existed for decades within the research, qualitative research with victims directly can allow for victims to indicate their experiences and their perception of what influenced their experiences. As previously mentioned, due to the nuance of these theories, other factors may be relevant and may be identified by victims.

## Conclusions

This study expands on the preexisting definitions of rape myths, toward an updated conceptualization of “rape myths,” including “real rape” and “real victim” and examines the relationships between these concepts and revictimization. The current analyses support the correlation between the two subscales of revictimization, behavior and emotion, and indicate that the revictimization behavior of questioning a victim's resistance correlates to their experience of revictimization emotion as predicted in the second hypothesis. The proposed relationships between “real rape,” “real victim,” and SWB as mediated by the two subscales of revictimization and predicted in the first and third hypotheses were not supported, but a significant direct relationship was found between “real victim” and SWB and time since the assault and SWB in both models. Overall, results evidence the need for more consistent approaches to the investigation of rape myths and how these contribute to the experiences of people reporting sexual assault. Further research with victims of rape and sexual assault directly may provide greater clarity on these concepts and relationships and allow for research that represents the experiences of those who chose to report sexual assaults.
